# Supplementary material for: Immunity from NK Cell Subsets Is Important for Vaccine-Mediated Protection in HPV+ Cancers
Source: Vaccines (Basel). 2024 Feb 17;12(2):206. doi: 10.3390/vaccines12020206 (PMC10892709; doi:10.3390/vaccines12020206)
Supplement: Supplementary file 1 [file vaccines-12-00206-s001.zip › vaccines-2819410-supplementary.pdf]

Supplemental Figures

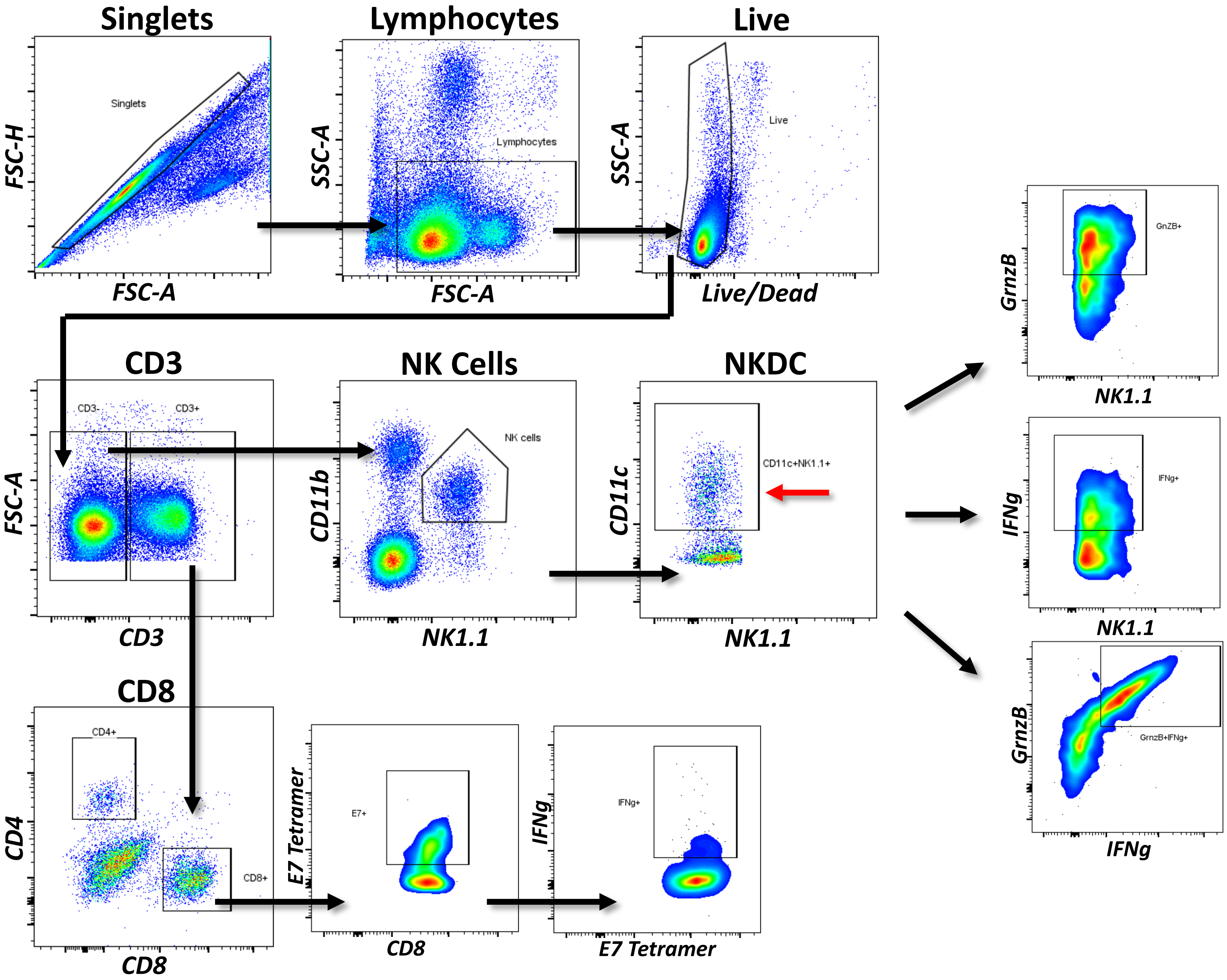

Supplemental Figure S1. Flow cytometry gating strategy for NKDC in mice.

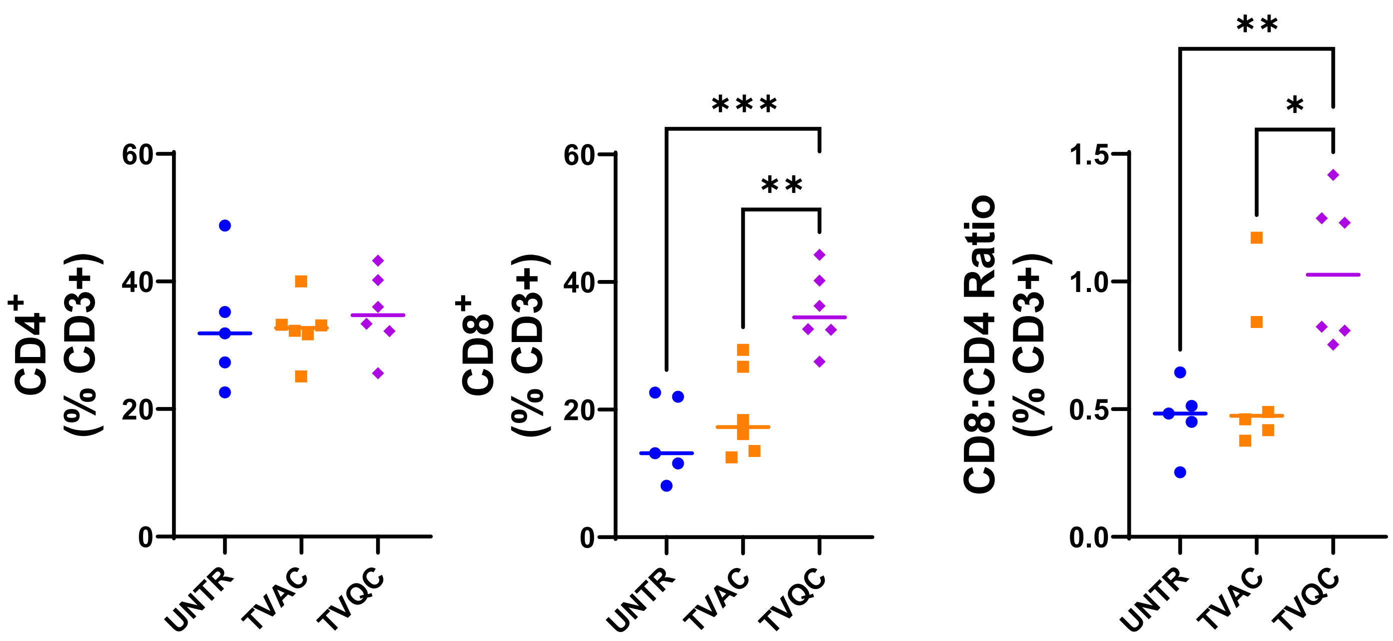

Supplemental Figure S2. Total CD4 and CD8 T cells. The frequencies of total CD4 and CD8 T cells, along with CD8:CD4 ratio, from TIL isolated from mEER oral tumors. Significance determined using Ordinary one-way ANOVA $*p<0.05$, $**p<0.005$ $***p<0.0005$ (n = 5-6 mice per group).

Supplemental Table S1. Antibodies for Flow Cytometry.

Antibody	Fluorophore	Supplier	Catalog #
CD3	BUV737	BD Bioscience	612803
CD11b	BV786	BD Bioscience	740861
NK1.1	PerCP-Cy5.5	BD Bioscience	551114
CTLA-4	APC-R700	BD Bioscience	565778
CD8	BUV395	BD Bioscience	563786
Granzyme B	BV421	Biolegend	396414
CD4	BV605	Biolegend	100548
CD45	BV650	Biolegend	103241
IFNg	BV711	Biolegend	505835
Foxp3	AF488	Biolegend	126406
CD11c	PE-Dazzle594	Biolegend	117348
PD-1	PE-Cy7	Biolegend	109110
Gr-1	APC-Fire750	Biolegend	108424
Arginase-1	PE	Life Technologies	12-3697-82
Live/Dead Aqua	BV510	Life Technologies	L34965
E7 Tetramer	APC	NIH	N/A
Fc Block	N/A	BD Bioscience	553142
